# A Single Paradigm for Implicit and Statistical Learning

**DOI:** 10.1111/tops.12439

**Published:** 2019-07-23

**Authors:** Padraic Monaghan, Christine Schoetensack, Patrick Rebuschat

**Affiliations:** ^1^ Department of English University of Amsterdam; ^2^ Department of Psychology Lancaster University; ^3^ School of Philosophy, Psychology, and Language Sciences Edinburgh University; ^4^ Department of Linguistics and English Language Lancaster University; ^5^ LEAD Graduate School and Research Network University of Tübingen

**Keywords:** Implicit learning, Statistical learning, Cross‐situational learning, Awareness, Language, Acquisition, Explicit instruction

## Abstract

Implicit learning generally refers to the acquisition of structures that, like knowledge of natural language grammar, are not available to awareness. In contrast, statistical learning has frequently been related to learning language structures that are explicitly available, such as vocabulary. In this paper, we report an experimental paradigm that enables testing of both classic implicit and statistical learning in language. The paradigm employs an artificial language comprising sentences that accompany visual scenes that they represent, thus combining artificial grammar learning with cross‐situational statistical learning of vocabulary. We show that this methodology enables a comparison between acquisition of grammar and vocabulary, and the influences on their learning. We show that both grammar and vocabulary are promoted by explicit information about the language structure, that awareness of structure affects acquisition during learning, and awareness precedes learning, but is not distinctive at the endpoint of learning. The two traditions of learning—implicit and statistical—can be conjoined in a single paradigm to explore both the phenomenological and learning consequences of statistical structural knowledge.

## Abstract 2

This article focuses on the implicit statistical learning of words and syntax. Monaghan, Schoetensack and Rebuschat introduce a novel paradigm that combines theoretical and methodological insights from the two research traditions, implicit learning and statistical learning. Their cross‐situational learning paradigm has been used in the statistical learning literature, while their measures of awareness have widely been used in implicit learning research. They illustrate how the two literatures can be conjoined in a single paradigm to explore implicit statistical learning.

## 1 Introduction

There has been a traditional distinction between implicit and statistical learning approaches to language research, not only in the theory, but also in the methods used and the types of language structures assessed in these fields. Implicit learning studies have tended to address unconscious compared to conscious knowledge of grammatical structures of sequences, whereas statistical learning studies have focused more on acquisition of words or simple local constraints in grammatical sequences ([Link] this issue; Gomez & Gerken, 1999; Perruchet & Pacton, 2006; Romberg & Saffran, 2010). Indeed, such a distinction between learning associated with grammar and learning associated with words has been underwritten by theories that assume only the latter process is related to statistical learning (e.g., Peña et al., 2002). Furthermore, learning words and grammar have been related to distinct memory systems ([Link] this issue; Schacter, 1987; Shanks & St. John, 1994), with vocabulary requiring the operation of explicit, or declarative, memory whereas syntax is acquired through implicit, or procedural, memory (Ullman, 2004, 2016), consistent with views of acquisition of sequencing constraints being acquired without awareness (e.g., Reber, 1967; Reber & Squire, 1994). Thus, the language learner can report what the words are in the language, but struggles to describe the grammatical structures inherent within the language.

Yet the distinction between acquisition of grammar and acquisition of vocabulary has been challenged by studies that suggest instead that similar statistical processes can apply to acquisition of both types of language structure (Bates & Goodman, 1997; Frost & Monaghan, 2016), though the order of acquisition of grammar and vocabulary remains an open question (Rebuschat, Monaghan, & Schoetensack, submitted).

Despite the implicit representation of grammar, explicit knowledge about grammatical structure has been found to promote language learning in adults (see Goo et al., 2015; Norris & Ortega, 2000; Spada & Tomita, 2010). A key question arises, then, about how knowledge of grammar can influence learning, both of the grammar itself, but also whether this knowledge can also promote learning of the vocabulary that the grammar contains. If so, then this certainly melds fields of implicit and statistical learning still more coherently. The paradigm that we describe in this paper illustrates the means by which both grammar and vocabulary learning can be explored, by simultaneously manipulating statistical learning processes associated with learning vocabulary but also implicit learning processes associated with grammar acquisition, with the possibility to introduce explicit information about grammatical structure into the learning paradigm and investigate the extent to which this knowledge penetrates acquisition of grammar as well as vocabulary learning.

The paradigm shows the convergence of implicit and statistical learning approaches, and involves an artificial language of intransitive sentences, comprising a noun and a verb along with a marker word that indicates the grammatical category of the following word (either a noun or a verb), with word order of nouns and verbs varying. Critically, sentences in the grammar are accompanied by two scenes each indicating an object undertaking an action, with the sentence referring to one scene. Participants, exposed to multiple instances, could learn through determining associations between certain objects and actions and certain nouns and verbs in the language. So, learning could proceed only if participants are sensitive to cross‐situational statistics, a hallmark of natural language learning situations (Monaghan, Mattock, Davies, & Smith, 2015; Scott & Fisher, 2012; Yu & Smith, 2007).

In addition to the cross‐situational statistical learning, participants can respond to learning about structure contained within the language—thus, learning that has been previously considered under the remit of implicit learning studies can also be determined and detected in this paradigm. Furthermore, information about the grammatical structure of the language can be manipulated to investigate how explicit knowledge can affect acquisition of grammar, as is often investigated in implicit and explicit learning studies, but its effect on vocabulary can also be examined—a methodological alignment of statistical with implicit learning approaches.

We next report two experimental studies exploring this paradigm providing insight into how the interface between explicit knowledge of language structure affects acquisition of vocabulary and grammar during learning. In Experiment 1, we investigated acquisition of vocabulary and grammar when explicit information about the grammar was or was not provided to participants prior to exposure to the artificial language. We predicted that participants who received explicit knowledge about the artificial language would be able to apply this knowledge of the syntactic structure of the language to acquire the relations between particular nouns and objects and verbs and actions. However, we were also interested in the trajectory of learning for when this knowledge provided an advantage. It could be that learning was promoted from the beginning of the learning, or it could be that the advantage of syntactic knowledge only occurred later in training when participants had also acquired some knowledge of potential associations between individual words and referents in the scenes. Thus, the study enabled us to address the issue of whether, for a language where both vocabulary and syntax are unknown in advance of learning, the learner’s derivation of syntactic knowledge preceded their vocabulary knowledge, thereby promoting acquisition of individual words, or whether syntax and vocabulary learning were instead mutually dependent (Abend, Kwiatkowski, Smith, Goldwater, & Steedman, 2017; Fisher et al., 2010; Landau & Gleitman, 1985).

Furthermore, we were also interested in the extent to which explicit syntactic knowledge of the language could emerge as a consequence of pure exposure to sentence‐scene correspondences, without explicit feedback. After exposure, we questioned participants on their knowledge of the language structure, and tracked the effect of this syntactic knowledge on participants’ performance in learning the sentence–scene correspondences. We predicted that, for some participants, they would acquire explicit knowledge of the language structure, and that this would relate to enhanced performance similar to those participants who were given the syntactic structure information from the outset of learning (e.g., Franco, Cleeremans, & Destrebecqz, 2016; Hamrick & Rebuschat, 2012). The results of these comparisons enable insight into the extent to which providing versus discovering language structure results in distinct patterns of behavior, with implications for the effectiveness of language instruction (N. C. Ellis, 2005a R. Ellis,2005b, 2015).

In the second experiment, we investigated in greater depth the point at which participants derived explicit knowledge of the syntactic structure of the language and their use of this knowledge in order to guide their acquisition of learning the word–referent mappings in the language. Experiment 1 tested how explicit knowledge of structure could affect learning, and whether awareness, as measured by retrospective verbal reports, affected performance during the acquisition of the language structure. In Experiment 2, we determined more precisely when explicit knowledge of structure emerged during training, and how this affected learning. In Experiment 2, we asked participants to report, on a trial by trial basis, what each classification decision was based on, with response options ranging from implicit to explicit sources of knowledge. Determining whether explicit knowledge preceded or followed an enhanced ability to acquire the language addresses the relation between vocabulary and syntax acquisition, and, more broadly, the relation between implicit and statistical learning.

## 2 Experiment 1: The effect of explicit instruction on learning grammar and vocabulary

### 2.1 Method

#### 2.1.1 Participants

Thirty‐one native speakers of English (19 female) were randomly assigned to either incidental (*n* = 15) or instructed (*n* = 16) exposure conditions. The majority of participants (30) were university students, and the mean age was 21.9 (*SD* = 2.5). Three participants reported speaking an additional native language other than English (Dutch, Punjabi, and Yoruba). Twenty‐nine participants indicated that they also had acquired one or more foreign languages, namely French (22), German (16), Spanish (9), Mandarin, Polish, and Urdu (each 1). One participant in the instructed group did not follow task instructions (taking phone calls during training), and data for this participant were excluded from the analyses. Participants received £15 for participating. Our target for the number of participants was 15 per condition, in accordance with other studies tested in our laboratory using cross‐situational language learning sufficient to reveal between‐condition differences in learning (Monaghan & Mattock, 2012, *N* = 15; Monaghan et al., 2015, *N* = 16).

#### 2.1.2 Materials

##### 2.1.2.1 Cross‐situational learning task

The materials for this task were taken from Monaghan et al. (2015). There were eight geometric shapes taken from Fiser and Aslin (2002). There were eight possible paths of motion for each object (bouncing, contracting, growing, hiding, rising, shaking, spinning, and swinging). There were 18 pseudowords: Sixteen were bisyllabic (each 900 ms in length) and two were monosyllabic (each 500 ms). The bisyllabic pseudowords were “content words” since they could either refer to the shapes or to motions that these objects could perform. The two monosyllabic pseudowords served as “function words” that indicated if the following content word referred to a shape or to a motion.

Eight of the bisyllabic words were paired with a shape each and the remaining eight bisyllabic words were paired with a motion. Pairings were randomized in six different versions to avoid biases linking particular words to shapes or motions.

##### 2.1.2.2 Questionnaires

A debriefing questionnaire was used to determine whether participants became aware of any patterns. Participants were first asked if they had noticed any rules or patterns in general. They were then asked if they noticed what type of word always followed the monosyllabic words (*tha* and *noo*). Participants were encouraged to write down their best guesses if they did not notice anything in particular. A background questionnaire asked for age, gender, educational background, native language(s), and any foreign languages studied by the participants.

#### 2.1.3 Procedure

Participants were tested individually in a quiet laboratory. They were randomly assigned to either an incidental or an instructed condition. Participants first completed the cross‐situational learning task, then the debriefing questionnaire (retrospective verbal reports), followed by the background questionnaire.

For the cross‐situational learning task, participants were told that they would see two scenes and hear a sentence and that their task was to choose which scene the sentence refers to. Both groups were then presented with an example where a rectangular shape performed a circling movement while the sentence “Tha trepier noo vinnoy” was played. The incidental group was informed that “trepier” referred to the shape and “vinnoy” to the circling movement. The instructed group was provided with explicit information about the function words: “Each sentence contains the name of an object and the name of its motion. The object name is always preceded by the word *tha*, and the motion name is always preceded by the word *noo*.” After seeing the example, they were informed that “tha trepier” referred to the shape and “noo vinnoy” to the circling movement. Participants in the instructed condition were reminded of the role of the function words halfway through the exposure task (during the break after block 6).

**Figure 1:**
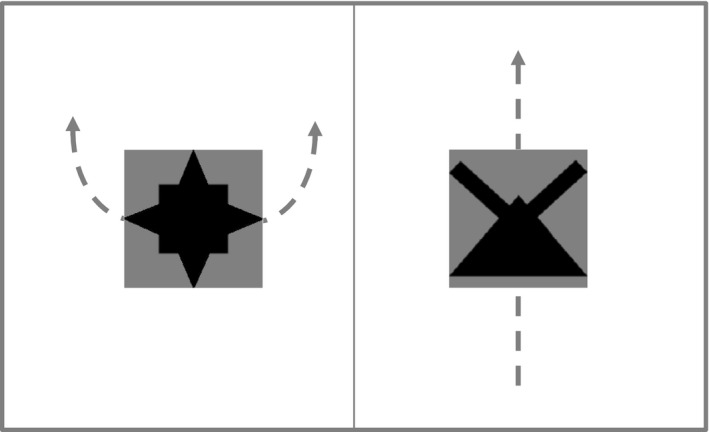
Example of learning trial. Participants are presented with two moving objects and a four‐word utterance (e.g., “Tha makkot noo pakrid”). In this example, the arrows indicate the movement paths of the objects. “Tha” and “noo” serve as function words, while “makkot” or “pakrid” either refer to the object or to its motion. Participants have to decide if the utterance describes the scene on the left or right of the screen.

For each trial, participants observed two scenes each comprising an object undergoing a motion. After 3 seconds, participants heard a sentence composed of two phrases (function word plus content word) with order of noun and verb phrases balanced across trials, so that the function words provided distributional information to indicate the content words’ roles (see Fig. 1 for example). Then, participants selected, as quickly and accurately as possible, whether the scene on the left or the right of the screen was described by the sentence with a keyboard press. After a pause of 500 ms, the next trial began. No feedback was provided on the accuracy of the response.

There were 12 training blocks, each containing 24 trials. Within each block, each motion occurred six times, and each word occurred three times. Position of the target scene was balanced within each block. Participants could take a short break after six training blocks.

Participants could solve the task by responding only to the noun–object or the verb–object pairing, and so two testing blocks were added after training. To test verb learning, participants were presented with two scenes containing a previously unseen object performing different motions. They then heard a single motion referring word and were required to select the scene described by the word. To test noun learning, participants viewed two stationary objects and heard a single object–referring word. There were 16 trials in total (one for each verb and noun), and no feedback was given on performance. The tests were administered twice in order to increase power for analysis. Since the task remained the same (choosing which scene matched the auditory stimulus), the test phase followed without a break from the training.

### 2.2 Results

Data and statistical analysis scripts in R are available at https://osf.io/2xzye/?view_only=24b19067006948a19ed03726290e8a63

#### 2.2.1 Performance on training blocks

**Figure 2:**
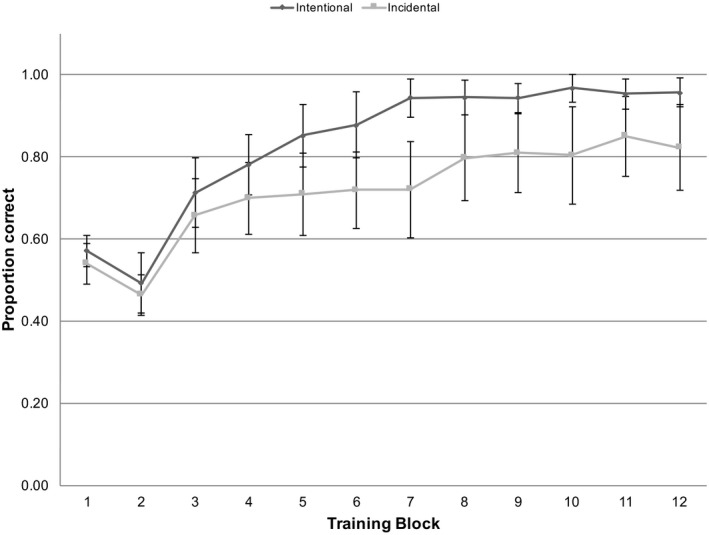
Mean proportion of correct pictures selected in each training block by participant. There were significant differences between the groups on blocks 5–10 and 12. Error bars represent 95% Confidence Intervals.

For proportion correct across all training blocks, the incidental group judged 0.72 (*SD* = 0.15) of trials correctly, which was significantly above chance, *t*(14) = 5.791, *p* < .001, *d* = 1.467, and the instructed group judged 0.83 (*SD* = 0.08) of items correctly, again above chance, *t*(14) = 15.641, *p* < .001, *d* = 4.125. In order to investigate individual variation in terms of learning, we used binomial tests to determine when participants first scored above chance (17 out of 24 correct) within each block, and remained above chance subsequently. Participants in the incidental group tended to perform above chance by block mean = 6.64, *SD* = 3.54, and the instructed group tended to perform above chance by block mean = 3.67, *SD* = 1.76. The difference between groups was significant, *t*(27) = 2.896, *p* = .007, Hedges’ *g* = 1.034 (Fig. 2).

In order to determine whether learning was different according to the incidental or instructed condition, we constructed a series of generalized linear mixed effects (GLME) models to test accuracy on each trial throughout training. GLME models enable variance associated with individual participants’ responses and with individual items to be taken into account in the analysis (random effects), and then the contribution of fixed effects of experimental manipulations in explaining variance can be observed over both participants and items. First, we constructed a model to predict whether a trial was correct or not with random effects of the participant, the object, and the action present in the target scene. Then, we added in variables to determine if these improved model fit using log‐likelihood tests. Random slopes for fixed effects were included for all random effects, except when the model failed to converge, in which case the model with the maximal random effects structure that converged was constructed (Barr, Levy, Scheepers, & Tily, 2013). To determine the effect size of the fit of each model, we calculated *R* ^2^, using the method reported in Nakagawa and Schielzeth (2013). There are two *R* ^2^ calculations that can be performed on GLME models: marginal *R* ^2^ is the proportion of variance in the data set explained by the fixed effects in the model, and conditional *R* ^2^ is the proportion of variance explained by both fixed and random effects. Nakagawa and Schielzeth’s (2013) method applies to models with random intercepts, and so we computed the *R* ^2^ values from models without random slopes.

Compared to the model containing only random effects, the effect of training block significantly improved model fit, χ^2^(1) = 35.489, *p* < .001, marginal *R* ^2^ = 0.289, conditional *R* ^2^ = 0.585, adding incidental versus instructed condition did not significantly improve fit, χ^2^(1) = 0.155, *p* = .694, marginal *R* ^2^ = 0.346, conditional *R* ^2^ = 0.585. The interaction between block and incidental versus instructed condition did improve model fit, χ^2^(1) = 4.727, *p* < .001, marginal *R* ^2^ = 0.449, conditional *R* ^2^ = .651. In Table 1, we report the final best‐fitting model.

**Table 1:** Best‐fitting model of proportion correct for Experiment 1, showing fixed effects

Fixed Effects	Estimate	*SE* Estimate	Z	*p*
(Intercept)	−0.541	0.146	−3.695	< .001
Block	0.495	0.064	7.708	< .001
Condition	0.189	0.172	1.099	.272
Block × Condition	−0.203	0.089	−2.281	.023

Number of observations: 8,640, Participants: 30, Actions: 8, Objects: 8. AIC = 7322.6, BIC = 7400.3, log‐likelihood = −3650.3.

R syntax: glmer(Accuracy ~ (1 + Block|Subject) + (1 | TargetAction) + (1 + instruction_condition|TargetPicture) + Block*instruction_condition, family = binomial )

We next tested for which of the training blocks the difference between incidental and instructed conditions was evident. We tested GLME models for single blocks, comparing the model fit for just the random effects to a model also containing incidental versus instructed condition. The results are summarized in Table 2. Instruction condition had a significant effect on accuracy in blocks 5–10 and block 12.

**Table 2:** Effect of condition for each block, with improvement in fit over random effects model tested with log‐likelihood comparison, and marginal and conditional *R* ^2^ of the model fit

Block	χ^2^(1)	*p*	Marginal *R* ^2^	Conditional *R* ^2^
1	0.684	.408	0.004	0.027
2	0.382	.536	0.003	0.079
3	0.738	.391	0.012	0.409
4	1.961	.161	0.034	0.404
5	3.965	.047	0.095	0.645
6	5.557	.018	0.142	0.668
7	10.021	.002	0.283	0.814
8	4.835	.028	0.153	0.842
9	5.327	.021	0.150	0.721
10	5.949	.015	0.213	0.883
11	2.598	.107	0.096	0.791
12	4.548	.033	0.150	0.808

We additionally tested whether language background—the number or the maximum proficiency of second languages spoken by participants (from 0 for no second languages, 1 for the beginner level, 2 for intermediate, and 3 for advanced)—improved model fit over the final model shown in Table 1. It did not: for number of languages spoken, χ^2^(1) = 0.440, *p* = .507, with marginal *R* ^2^ = 0.452, conditional *R* ^2^ = 0.651; for maximum proficiency level, χ^2^(1) = 0.145, *p* = .703, marginal *R* ^2^ = 0.450, conditional *R* ^2^ = 0.651. The marginal and conditional *R* ^2^ values accounted for very small increases in variance compared to the model in Table 1.

#### 2.2.2 Performance on test blocks

For the test trials that determined whether learning was based on nouns or verbs, or both, in the incidental group, participants scored 0.84 (*SD* = 0.19) in the noun test and 0.84 (*SD* = 0.20) in the verb test, both significantly better than chance, *t*(14) =  6.931, *d* = 1.789, *t*(14) = 6.584, *d* = 1.700, respectively, both *p* < .001. In the instructed group, participants scored .95 (*SD* = 0.08) in the noun test and 0.97 (*SD* = 0.06) in the verb test, significantly above chance for both word types, *t*(14) = 21.786, *d* = 5.625, *t*(14) = 30.338, *d* = 7.833, respectively, both *p* < .001.

GLME models indicated that whether the testing was the first or the second block had no significant effect, χ^2^(1) = 0.228, *p* = .633, marginal *R* ^2^ = 0.001, conditional *R* ^2^ = 0.762; nor was there a significant effect of nouns or verbs, χ^2^(1) = 0.001, *p* = .975, marginal *R* ^2^ < 0.001, conditional *R* ^2^ = 0.761. There was a significant effect of instructed or incidental group, χ^2^(1) = 5.840, *p* = 0.016, marginal *R* ^2^ = 0.167, conditional *R* ^2^ = 0.749. There were no significant interactions. Language background did not significantly improve model fit: For number of languages spoken, χ^2^(1) = 0.089, *p* = .765, with marginal *R* ^2^ = 0.169, conditional *R* ^2^ = 0.750; for maximum proficiency level, χ^2^(1) = 0.243, *p* = .622, marginal *R* ^2^ = 0.172, conditional *R* ^2^ = 0.750. Language background did not significantly improve model fit for any of the other tests Experiments 1 or 2, so we do not mention them further. The final model including the effect of group is shown in Table 3.

**Table 3:** Final model for testing performance for Experiment 1, showing fixed effects

Fixed Effects	Estimate	*SE* Estimate	Z	*p*
(Intercept)	3.968	0.562	7.056	< .001
Condition	−1.628	0.642	−2.535	.011

Number of observations: 960, Participants: 30, Objects: 9 (including novel unnamed object). AIC = 531.3, BIC = 550.8, log‐likelihood = −261.7.

R syntax: glmer(Testaccuracy ~ (1|Subject) + (1|TargetNoun) + instruction_condition, family = binomial )

#### 2.2.3 Retrospective verbal reports

We coded the verbal reports to determine whether participants became aware of any patterns in the language. As predicted, all participants in the instructed group were aware of the function words and of the role they played. Seven subjects in the incidental condition also became fully aware of both function words and of the types of words with which they were associated. No participant reported trying to intentionally learn the function words as a strategy; thus, this grammatical knowledge was acquired incidentally, as a side‐effect of completing the exposure task. In order to determine whether awareness of the structure in the incidental group related to performance during training, we compared the performance of aware and unaware participants of the incidental group on the cross‐situational learning task. The online response design of our study also enabled us to determine at what point during training such awareness might have first had an influence on performance.

In terms of overall accuracy, the unaware participants (*n* = 7) judged 0.66 (*SD* = 0.11) of trials correctly, which was significantly above chance, *t*(6) = 3.848, *p* = .009, *d* = 1.455, while the aware participants (*n* = 7) judged 0.80 (*SD* = 0.15) of items correctly, *t*(6) = 5.292, *p* = .001, *d* = 2.000. Binomial tests of when participants first scored above chance and remained above chance until the end of training demonstrated that the unaware participants consistently performed above chance from block 8 (*M* = 7.50, *SD* = 2.43) and the aware participants from block 5 (*M* = 5.14, *SD* = 3.76). However, the difference between groups was not significant, *t*(10.326) = 1.314, *p* > .05, Hedge’s g = 0.698.

**Figure 3:**
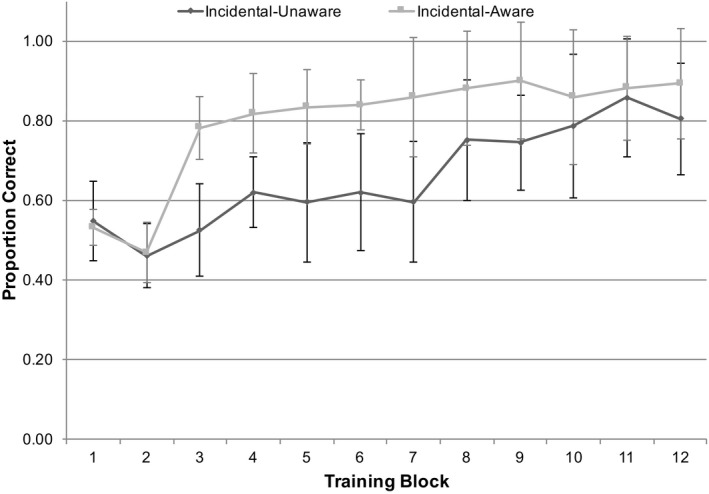
Proportion of correct responses in each training block for aware and unaware participants of the incidental group. Performance in blocks 3–7 differed significantly between groups. Bars represent 95% CIs for a by‐items analysis of accuracy.

We tested whether awareness affected learning by comparing GLME models. For performance in the incidental condition, there was a significant improvement in fit by adding block to the model, χ^2^(1) = 229.49, *p* < .001, marginal *R* ^2^ = 0.107, conditional *R* ^2^ = 0.511. There was also a significant improvement in fit by distinguishing aware and unaware participants, χ^2^(1) = 3.947, *p* = .047, marginal *R* ^2^ = 0.264, conditional *R* ^2^ = 0.512. The interaction between block and awareness was not significant, χ^2^(1) = 2.786, *p* = .095, marginal *R* ^2^ = 0.277, conditional *R* ^2^ = 0.523, and awareness was not related to better performance at the end of training, with no significant improvement in fit by adding the awareness group to a random effects model at the final training block, χ^2^(1) = 1.082, *p* = .298, marginal *R* ^2^ = 0.077, conditional *R* ^2^ = 0.761. Fig. 3 shows the results for aware and unaware participants during training.

For the test of noun and verb learning after training, awareness did not significantly improve model fit, χ^2^(1) = 0.615, *p* = .433, marginal *R* ^2^ = 0.037, conditional *R* ^2^ = 0.739, indicating that by the end of training, word learning was similar across aware and unaware participants.

### 2.3 Discussion

Experiment 1 demonstrated that classic implicit learning and statistical learning approaches can be combined in a single methodology. We showed that the effectiveness of cross‐situational learning—a task that has been used to test statistical learning in acquisition of language—can be modulated by explicit knowledge of the language structure to be acquired, consistent with many other studies of the benefit of syntactic information for language learning (Goo et al., 2015; Spada & Tomita, 2010). This alignment of approaches enables links between theories of statistical learning with theories of effects of explicit structural knowledge. For instance, the advantage of explicit knowledge about syntactic structure appeared only to able to promote learning once a proportion of the words had been acquired in the language, in accord with Gleitman’s (1990) view of syntactic knowledge applying to learning only once sufficient vocabulary had been acquired in the language. However, tracking the trajectory of learning through training of the participants in the incidental group that independently learned about the structure of the syntax at the end of training indicates a slightly different picture. As shown in Fig. 3, participants who later became aware of the syntactic structure were outperforming those that remained unaware in learning the cross‐situational statistics early in training. Interestingly, emerging awareness did not distinguish performance at the end of the task (as indicated in the final block of training and the vocabulary testing), but it did show a distinction in performance during the acquisition of the cross‐situational statistics task.

Awareness of the acquired knowledge was only measured at the end of training (by means of retrospective verbal reports). Hence, we have no direct evidence of the point at which explicit knowledge of the language structure was first derived. The steep increase in vocabulary acquisition, as shown in Fig. 3 for the incidental group that gained explicit knowledge, may have precipitated understanding of the syntactic structure, or alternatively emerging explicit knowledge may have resulted in the increase. We do not yet know the causative direction of this effect. Experiment 2 addresses this issue.

## 3 Experiment 2: The emergence of explicit knowledge during cross‐situational learning

This study was similar to the incidental exposure condition of Experiment 1, except that for each trial during training, participants were asked about their awareness of the language, responding according to whether their decision was based on a guess, intuition, recollection of previous trials, or knowledge based on a rule (Subjective measures of awareness; Dienes & Scott, 2005; Rebuschat & Williams, 2012; see Rebuschat, 2013, for review of this approach). We predicted that initially most responses would be based on the more implicit categories (guess and intuition), but that during training the proportion of responses based on the more explicit categories (recollection and rule knowledge) would increase. In addition to these subjective measures of awareness, we also measured awareness by means of retrospective verbal reports, as in Experiment 1.

If awareness precedes learning, we predicted that there would be a correspondence between accuracy of responses for each type of explicit knowledge category, such that rule knowledge responses would be most accurate, then recollection, intuition, and then guesses. If awareness follows learning, then we predicted increasing rates of rule knowledge responses without these relating directly to accuracy.

### 3.1 Method

#### 3.1.1 Participants

Twenty‐two university students (18 female) who were English native speakers received 10 GBP for participating. The mean age was 20.23 (*SD* = 3.01).

#### 3.1.2 Materials

The scenes and language were identical to those used in Experiment 1.

#### 3.1.3 Procedure

The procedure was identical to the incidental exposure condition of Experiment 1 except for two details. First, the description of the trial prior to training (present in Experiment 1) was not given. Second, after making a decision about the scene described by the sentence in each training trial, participants were then asked to make an additional judgment in order to report the basis of their decision by selecting one of four adjacent keys (labeled “G” for guess, “I” for intuition, “R” for recollection, “K” for rule knowledge) on a keyboard. Participants were asked to select the guess category if their decision was based on a true guess; that is, they might as well have flipped a coin. Participants were instructed to select the intuition category if they felt their decision was correct but they could not explain why; that is, they just followed a hunch. In contrast, participants were asked to select the recollection category if the decision was based on conscious recollection of specific sequences (or parts of sequences) that they have heard before, while they should select the rule knowledge category if they followed a conscious (verbalizable) rule when making the decision. The actual wording of the instructions can be found in the Supplementary Materials online. Following Dienes and Scott (2005), decisions based on guessing and intuition were linked to implicit knowledge, while decisions based on conscious recollection and rule knowledge were associated with explicit knowledge; full instructions and scale description are described in the Supporting Information.

### 3.2 Results and discussion

#### 3.2.1 Performance during training and testing blocks

Over all blocks, participants judged 0.79 (*SD* = 0.07) of trials correctly, which was significantly above chance, *t*(18) = 17.287, *p* < .001, *d* = 4.143. Participants performed above chance in all blocks, *t*(21) > 2.756, *p* < .012, *d* > 1.175, except for block 2, *t*(21) = 0.344, *p* = .734, *d* = 0.147.

**Figure 4:**
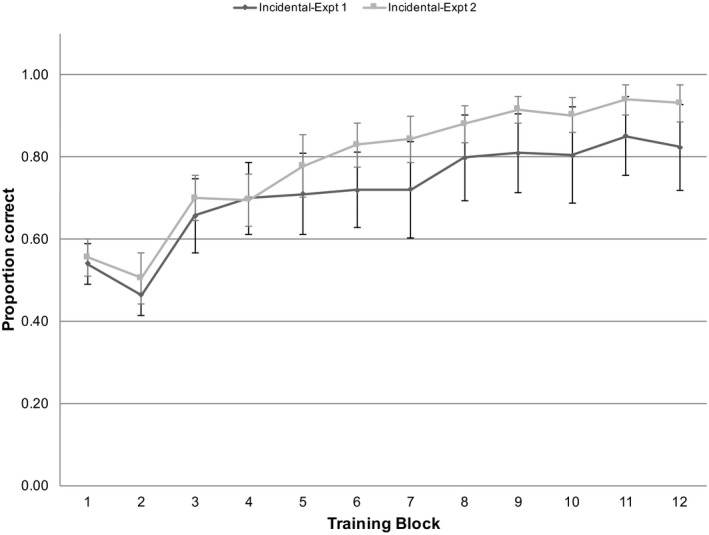
Proportion correct by training block in Experiment 2, compared to incidental exposure condition of Experiment 1. Error bars represent 95% CIs.

We next tested whether performance improved with training block using GLME models. Adding block to a model containing only random effects significantly improved model fit, χ^2^(1) = 27.881, *p* < .001, marginal *R* ^2^ = 0.299, conditional *R* ^2^ = 0.502. Fig. 4 shows the performance by block, in comparison to the incidental exposure condition of Experiment 1.

For the tests of noun and verb learning, participants judged 0.86 (*SD* = 0.20) of trials correctly in the noun test and 0.82 (*SD* = 0.18) of trials in the verb test. Again, performance was significantly above chance (noun test: *t*(21) = 8.452, *d* = 1.800, verb test: *t*(21) = 8.170, *d* = 1.778, both *p* < .001). There was no significant improvement in fit including first or second test in a GLME model, χ^2^(1) = .773, *p* = .379, marginal *R* ^2^ = 0.004, conditional *R* ^2^ = 0.598, nor was there a significant improvement in fit when considering the effect of whether the noun or the verb was being tested, χ^2^(1) = 1.741, *p* = .187, marginal *R* ^2^ = 0.008, conditional *R* ^2^ = 0.601; thus, participants acquired both nouns and verbs to a similar degree of accuracy.

#### 3.2.2 Subjective measures of awareness

For the different categories of knowledge, participants were correct for 0.53 (*SD* = 0.11) of trials when they believed they were guessing, not significantly different than chance, *t*(16) = 1.125, *p* = .277, *d* = 0.273. Accuracy was greater than chance for other response types: for intuition, mean = 0.67 (*SD* = 0.16), *t*(16) = 5.002, *p* < .001, *d* = 1.063, for recollection, mean = 0.79 (*SD* = 0.15), *t*(15) = 9.259, *p* < .001, *d* = 1.933, and for rule knowledge, mean = 0.93 (*SD* = 0.23), *t*(17) = 8.187, *p* < .001, *d* = 1.870. Across the 12 training blocks, Table 4 shows proportion of responses by response category, which indicated a gradual shift from implicit responses (guess and intuition) to explicit responses (recollection and rule knowledge). A GLME model on accuracy with block as fixed effect was improved significantly in fit by adding whether the response was implicit or explicit, χ^2^(1) = 164.28, *p* < .001, marginal *R* ^2^ = 0.446, conditional *R* ^2^ = 0.561.

**Table 4:** Mean and *SD* accuracy, and proportion of responses by category of response over the 12 blocks of training

		Block											
		1	2	3	4	5	6	7	8	9	10	11	12
Guess	M	0.51	0.48	0.52	0.54	0.56	0.63*	0.66*	0.52	0.68^+^	0.61	0.56	0.48
	*SD*	0.23	0.20	0.33	0.26	0.30	0.22	0.27	0.28	0.35	0.39	0.43	0.39
	Proportion	0.43	0.37	0.31	0.26	0.27	0.23	0.22	0.16	0.13	0.12	0.08	0.10
Intuition	M	0.57	0.55	0.59	0.57	0.72**	0.76**	0.80**	0.82**	0.82**	0.76**	0.78**	79**
	*SD*	0.20	0.19	0.23	0.22	0.24	0.25	0.21	0.24	0.19	0.21	0.25	0.22
	Proportion	0.38	0.35	0.34	0.40	0.30	0.30	0.28	0.27	0.22	0.23	0.22	0.18
Recollection	M	0.58	0.52	0.74**	0.72*	0.80**	0.92**	0.91**	0.90**	0.90**	0.90	0.93**	0.87**
	*SD*	0.25	0.28	0.24	0.33	0.24	0.15	0.14	0.17	0.15	0.16	0.15	0.20
	Proportion	0.18	0.26	0.31	0.23	0.25	0.26	0.25	0.23	0.28	0.22	0.23	0.23
Rule knowledge	M	1.00	0.17	0.97**	1.00**	0.95**	0.99**	0.98**	1.00**	0.98**	0.99**	0.99**	0.99**
	*SD*		0.33	0.06	0.00	0.14	0.05	0.05	0.02	0.03	0.04	0.04	0.03
	Proportion	0.00	0.02	0.04	0.11	0.18	0.22	0.26	0.34	0.37	0.43	0.47	0.49

Note:

^+^ *p* < .10, *p < .05, **p < .001.

In order to determine whether responses were predicted not only by current implicit or explicit response, we determined whether the most recent previous training trial that contained the same object had an implicit or an explicit response and if that response could predict current accuracy in addition to current response type. We also determined whether the most recent previous training trial containing the same action had an implicit or explicit response. As there was a relation between explicit judgments and accuracy, we constructed a GLME model with block, implicit/explicit response on the current trial, and accuracy of the previous trial containing the same object and accuracy of the previous trial containing the same action. We then determined whether adding implicit/explicit judgment to this model predicted additional variance. We found that it did, χ^2^(2) = 9.250, *p* = .009, marginal *R* ^2^ = 0.298, conditional *R* ^2^ = 0.502. Thus, participants were able to generate explicit knowledge about the structure prior to accuracy on the next trial containing similar information.

#### 3.2.3 Retrospective verbal reports

The analysis indicated that 17 out of 19 participants were aware of the function words and of the role they played by the end of Experiment 2. This was significantly differently distributed than for the incidental condition of Experiment 1, χ^2^(1) = 6.332, *p* = .012, Cramer’s V = 0.035, suggesting that the requirement to make a decision about the source of knowledge during training resulted in greater explicit awareness by the end of the study, but that this was only a small effect size. Such a result is consistent with observations of a learning advantage for an attribution decision about knowledge at each trial found in a Reber‐style artificial grammar learning task (Ivanchei & Moroshkina, 2018).

## 4 General discussion

In this paper, we have adapted the cross‐situational learning paradigm, which has been widely used to investigate the statistical mechanisms available to learners in acquiring language, to bridge the distinct traditions of implicit and statistical (language) learning. In adapting the paradigm, we have carefully manipulated exposure conditions (incidental vs. instructed) and included measures of awareness (verbal reports and subject measures), which is typically gathered in implicit learning research but not in statistical learning research (see Arciuli et al., 2014; Batterink et al., 2015; Franco, Cleeremans, & Destrebecqz, 2016; Hamrick & Rebuschat, 2012; Kachergis et al., 2010, for exceptions). By incorporating syntactic structure, in addition to word to object (Yu & Smith, 2007) and word to action (Scott & Fisher, 2012) mappings, this enabled us to determine the extent to which knowledge of the syntactic structure emerged, and could be manipulated during language development. Experiment 1 showed that instruction about the structure of the language could influence the statistical processing by improving acquisition of word to referent mappings across situations. However, this effect exerted itself during acquisition—by the end of acquisition, performance was similar between groups provided with explicit knowledge compared to those that were not given this information in advance. Experiment 1 also demonstrated that this paradigm could reveal differences during learning that related to whether participants had determined the language structure themselves as a consequence of performing the task, compared to those that had not. Intriguingly, once again those that developed emerging explicit knowledge performed differently during the task. However, this difference in their ability to solve the task was not detectable anymore by the end of the task, highlighting the importance of learning tasks that permit the online tracking of performance.

Experiment 2 addressed a key question in language acquisition research about when syntactic knowledge can be used in concert with vocabulary information. Experiment 2 asked participants to give information on the source of their decision at each cross‐situational learning training trial. We found that explicit knowledge about the language structure was able to predict accuracy of the participants learning the next time a similar word to object or word to action mapping was experienced. Thus, explicit knowledge preceded application of that knowledge for the task.

In terms of future directions, we have shown how statistical learning—beyond acquisition of adjacent or non‐adjacent dependencies in sequences—can be extended effectively to studies that investigate statistical correspondences across situations that are separate instances of learning. Furthermore, we have shown how classic tools in implicit learning research (e.g., measures of awareness and manipulation of exposure condition) can address foundational questions in the study of statistical learning of language. For instance, we have demonstrated how provision of explicit information about the structure of the language can affect statistical learning—during the process, if not the endpoint, of acquisition of the language. We have also shown how the individual’s determination of the source of their knowledge of the language structure—implicit or explicit—can precede accurate learning of mappings between the language and objects and actions in the world. Further work in this area should address the types of syntactic structures that can be acquired prior to, or simultaneously with, development of vocabulary knowledge. In the current study, we have only investigated learning of nouns and verbs in a simple intransitive sentence. Whether participants can align knowledge of more complex syntactic structures—such as hierarchical dependencies—with acquisition of vocabulary across learning situations would enable us to address how implicit and explicit knowledge about structure can scale up to support learning of complex, more realistic phrase structure grammars that occur in natural language.

## Supporting information


**Data S1.** Instructions for Experiment 2.
