# Supplementary material for: A Single Paradigm for Implicit and Statistical Learning
Source: Top Cogn Sci. 2019 Jul 23;11(3):536–54. doi: 10.1111/tops.12439 (PMC6852402; doi:10.1111/tops.12439)
Supplement: Supplementary file 1 — Data S1. Instructions for Experiment 2. [file TOPS-11-536-s001.pdf]

Instructions for Experiment 2

Monaghan, Schoetensack and Rebuschat (under review)

Screen 1:

In this study, you will see two scenes on the screen and hear a sentence.

For each trial, you have to do two things:

1. **Decide which of the two scenes is described by the sentence.**
    - Press 1 if the sentence refers to the left scene.
    - Press 2 if the sentence refers to the right scene.
    - Try to respond as quickly and accurately as possible. Simply pick whatever comes to your mind first!
2. **Report what your decision was based on: Guess, intuition, recollection, or rule knowledge.**

Press space bar to continue.

Screen 2:

To report the basis of your decision, please use the following categories:

Press G for GUESS: Your decision was based on a true guess, i.e. you might as well have flipped a coin.

Press I for INTUITION: Your decision was based on intuition, i.e. you feel that your decision is correct but you have no idea why. You just followed a hunch.

Press R for RECOLLECTION: Your decision was based on recollection of specific sequences (or parts of sequences) you have heard before that are similar.

Press K for RULE KNOWLEDGE: Your decision was based on rule knowledge, i.e. you followed a rule when making the decision and you are able describe the rule at the end of the experiment.

Press space bar to continue.

Screen 3:

You are now ready to start the experiment. This part of the study lasts about 40 minutes. You will be able to take a quick break halfway through.

Remember:

1. Try to decide which scene the sentence refers to as quickly and accurately as possible. Simply pick whatever comes to your mind first.
2. When reporting the basis of your decision...
    - Only use the guess category if you really have no idea.
    - If you have a hunch that you might be right, you should pick intuition.
    - In the case of recollection, please use this category if you are basing your decision on specific previous examples that you consciously remember.
    - Only use the rule category, if you are able to tell us what the rule was at the end of the experiment.

If you have any questions, please ask now. If not, press any key to begin.
